# Supplementary material for: Reduced mood variability is associated with enhanced performance during ultrarunnning
Source: PLoS One. 2021 Sep 16;16(9):e0256888. doi: 10.1371/journal.pone.0256888 (PMC8445466; doi:10.1371/journal.pone.0256888)
Supplement: S1 File — (DOCX) [file pone.0256888.s001.docx]

**S1 Analysis of all 30 participants who completed all within-race measurements (N=30).**

Data from the 30 participants for whom we had full datasets for the within-race measurements were analysed with a 4 (Measurement Point: 0 km, 34 km, 68 km, 103 km (Finish)) x 6 (Mood Factor: anger, confusion, depression, fatigue, tension, vigour) repeated measures ANOVA. There was no significant main effect of Measurement Point; *f*_(3,79)_ = 2.39, *p* = .074, η²p = .076. There was a large and statistically significant main effect of Mood; *f*_(5,145)_ = 71.06, *p* < .001, η²p = .71, and a significant Measurement Point x Mood interaction; *f*_(15, 435)_ = 26.46, *p* < .001, η²p = .48. Analysis of simple main effects are shown in Table S1.1. The factors of anger and fatigue increased significantly as the race progressed, whereas the factors of tension and vigour decreased significantly as the race progressed. Fig S1.1 illustrates these effects.

| **Table S1.1: Simple Main Effects - Measurement Point** | | | | | | | | | | | |
| --- | --- | --- | --- | --- | --- | --- | --- | --- | --- | --- | --- |
| **Level of Mood Factor** | | **Sum of Squares** | | **df** | | **Mean Square** | | **F** | | **p** | |
| Anger |  | 16.492 |  | 3 |  | 5.497 |  | 3.374 |  | 0.022 |  |
| Confusion |  | 11.667 |  | 3 |  | 3.889 |  | 2.596 |  | 0.058 |  |
| Depression |  | 6.067 |  | 3 |  | 2.022 |  | 1.006 |  | 0.394 |  |
| Fatigue |  | 788.200 |  | 3 |  | 262.733 |  | 46.668 |  | < .001 |  |
| Tension |  | 191.900 |  | 3 |  | 63.967 |  | 13.326 |  | < .001 |  |
| Vigour |  | 658.433 |  | 3 |  | 219.478 |  | 26.518 |  | < .001 |  |
|  | | | | | | | | | | | |
| *Note.*  Type III Sum of Squares | | | | | | | | | | | |


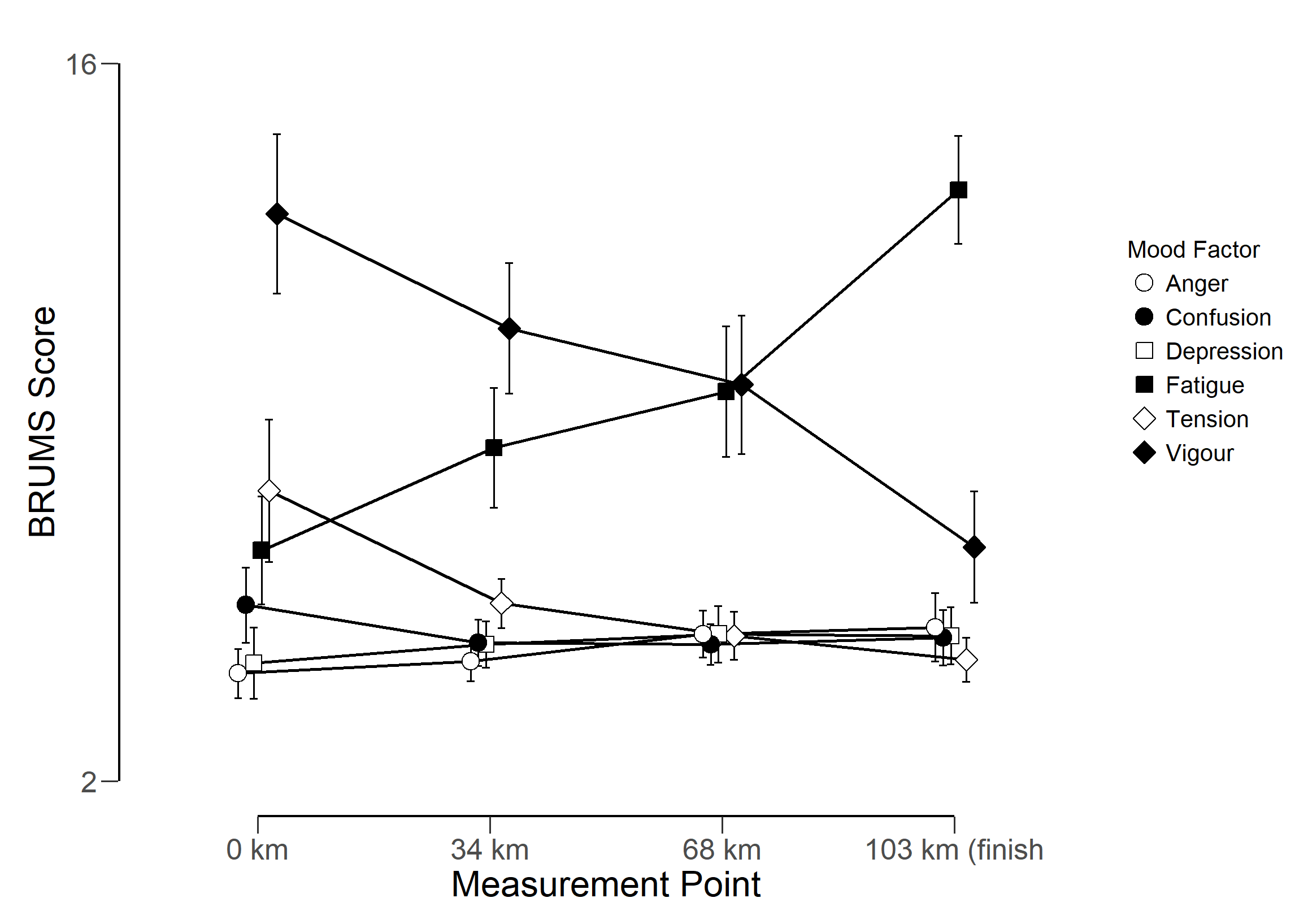


**Fig S1.1. Mean BRUMS scores for each mood factor at each measurement point.** Error bars show 95% confidence intervals.

The significant simple main effects were further explored using paired samples t-tests comparing adjacent measurement points. For anger there were no statistically significantly contrasts. Fatigue significantly increased between 0 km and 34 miles (*t*_(29)_ = 3.42, *p* < .01, *d* = .64) and between 68 km and 100 km (*t*_(29)_ = 6.29, p < .01, *d* = 1). However, the increase between 34 and 68 km was not statistically significant when the correction for multiple comparisons was applied (*t_(_*_29)_ = 2.09, *p* = .045, *d*= .38). Vigour decreased between 0 and 34 km (*t*_(29)_ = 4.28, p < .01, *d* = .8) and between 68 km and Finish (*t*_(29)_ = 4.56, p < .01, *d* = 83). However, there was no statistically significant change between 34 and 68 km (*t*_(29)_ = 1.58, p = .13, *d* = .28). Tension significantly decreased between 0 km and 34 km (*t*_(25)_ = 3.28, p < .01, *d* = .60). Neither of the other comparisons were statistically significant after the corrections for multiple comparisons was applied.
