# Supplementary material for: Reduced mood variability is associated with enhanced performance during ultrarunnning
Source: PLoS One. 2021 Sep 16;16(9):e0256888. doi: 10.1371/journal.pone.0256888 (PMC8445466; doi:10.1371/journal.pone.0256888)
Supplement: Attachment — Submitted filename: Response to reviews r2.docx [file pone.0256888.s003.docx]

Response to Reviewers

Dear Dr Tauler

Thank you for the opportunity to make these final revisions to our manuscript. The item by item response to the revisions can be found below

Many thanks

Dan Smith & Paul Burgum

The following comments should be addressed:

- Revise reference inserts format when more than two manuscripts are included. For example, in line 60-61, replace [1,2,3] to [1-3], or in line 78 replace to [11-18].

References have been amended accordingly. We have not changed the reference on line 84 because the citations are non-consecutive

- Lines 197-199. Please include a proper “Statistical analysis” subsection, including **all** information regarding any statistical analysis performed, software used, etc., at the end of the “Methods” section. Information such as the one reported in lines 203-204 should be also included in this section rather than in the Results section.

We have included a Statistical analysis section and moved all the details of the analyses to this section.

- Lines 144-145. While this comment is adequate, “kilometers” have appeared in the manuscript from the abstract. Therefore, the full name or the abbreviation of kilometers could be removed from these lines as this unit has been widely used previously in the manuscript.
- Decimal places should be carefully revised. In my opinion one decimal place for age is enough and more adequate.  Furthermore, the same decimal places in the mean and in the SD should be included for the same parameter (for example in Table 1, there’s no sense to include two decimals for the SD of parameters with no decimal places in the mean). In addition if the measure does not allow any decimal places they should not be used.

Decimal places have been revised as suggested

- Table 1. Parameters and units should be clarified. For example: Previous Ultra's over 80km (n). The use of “PW” could be avoided as the complete form “per week” could be included. Actually, the term “training level (km/week” could be used, with a proper explanation in the foot-note of the table.

Parameter names have been revised to make them more informative and appropriate footnotes added

- Lines 262-278. Describe this exploratory analysis previously, in the Methods section (statistical analysis), and report only the results in this “Results” section.

This section has been moved, as required

- Figure legends. Show the symbol used rather than descriptions such as “stars” or “white discs”.

Legends revised

- Uniformize expressions such as 49km (for example in line 269) and 35.4 km (line 289). In my opinion a space should be left between the number and the unit (49 km, 35.4 km).

The manuscript has been revised to ensure a space has been left between numbers and units.

- Conclusion section should be shortened without loosing significance. For example, the first sentence can be removed as it is such as the fourth time the aim of the study is stated (abstract, introduction, beginning of the discussion and conclusions). Comparisons with other studies can be also removed as they are discussed properly in the discussion section (or instead, moved to the discussion section).

The conclusion has been revised as suggested make it more concise.
